# Supplementary figures and images for: Exploring Honeybee Abdominal Anatomy through Micro-CT and Novel Multi-Staining Approaches
Source: Insects. 2022 Jun 18;13(6):556. doi: 10.3390/insects13060556 (PMC9224579; doi:10.3390/insects13060556)

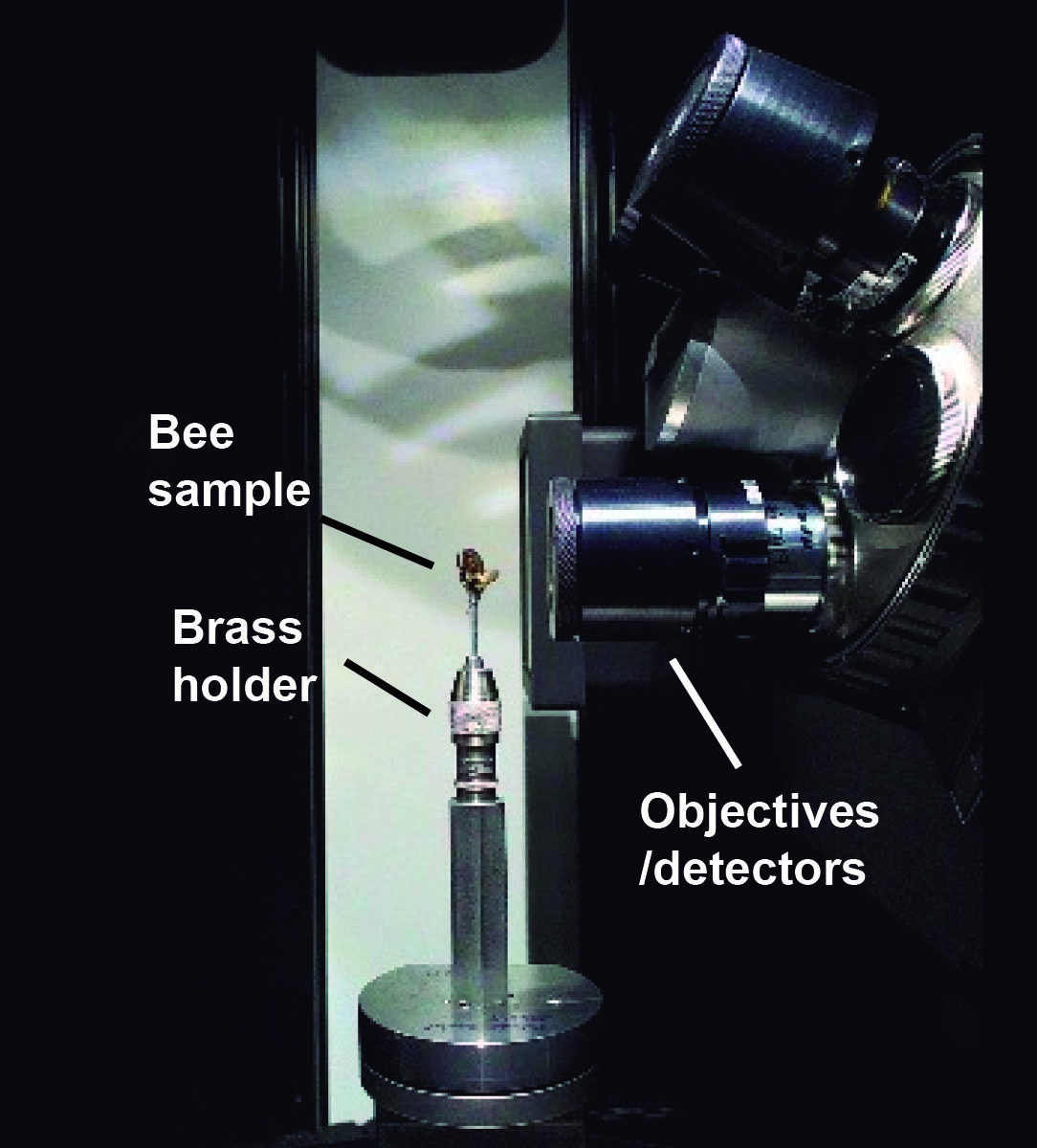

Supplement: Supplementary file 1 [file insects-13-00556-s001.zip › Supplementary Figure S1.jpg]

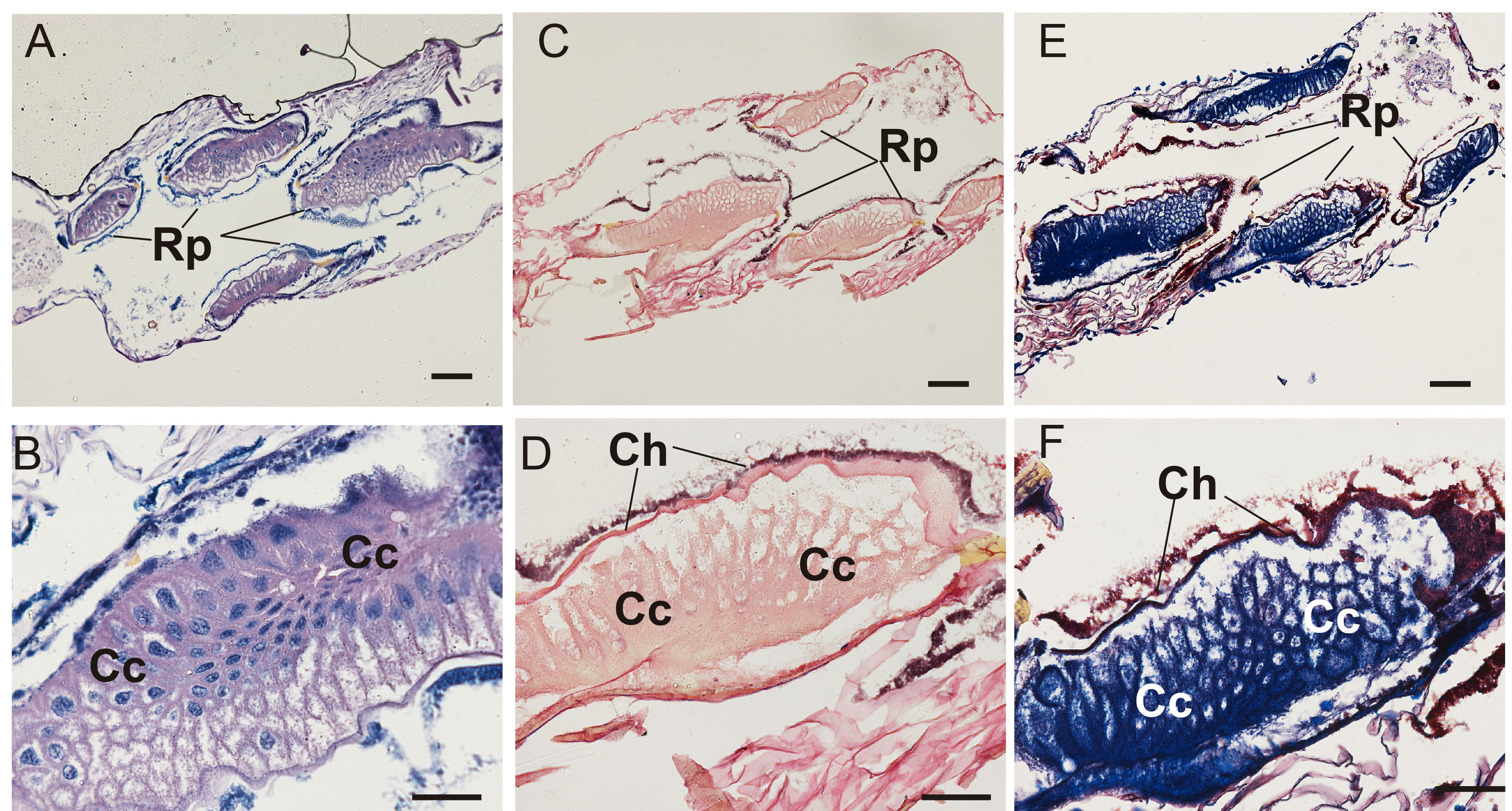

Supplement: Supplementary file 1 [file insects-13-00556-s001.zip › Supplementary Figure S2.jpg]
